# Identification of Genes Related to Immune Infiltration in the Tumor Microenvironment of Cutaneous Melanoma

**DOI:** 10.3389/fonc.2021.615963

**Published:** 2021-05-28

**Authors:** Rujia Qin, Wen Peng, Xuemin Wang, Chunyan Li, Yan Xi, Zhaoming Zhong, Chuanzheng Sun

**Affiliations:** ^1^ Department of Head and Neck Surgery Section II, The Third Affiliated Hospital of Kunming Medical University, Yunnan Cancer Hospital, Kunming, China; ^2^ Department of Medical Oncology, The First Affiliated Hospital of Kunming Medical University, Kunming, China

**Keywords:** cutaneous melanoma, tumor microenvironment, ESTIMATE, CIBERSORT, protein–protein interaction, weighted gene co-expression network analysis, tumor- infiltrating immune cells, prognosis

## Abstract

Cutaneous melanoma (CM) is the leading cause of skin cancer deaths and is typically diagnosed at an advanced stage, resulting in a poor prognosis. The tumor microenvironment (TME) plays a significant role in tumorigenesis and CM progression, but the dynamic regulation of immune and stromal components is not yet fully understood. In the present study, we quantified the ratio between immune and stromal components and the proportion of tumor-infiltrating immune cells (TICs), based on the ESTIMATE and CIBERSORT computational methods, in 471 cases of skin CM (SKCM) obtained from The Cancer Genome Atlas (TCGA) database. Differentially expressed genes (DEGs) were analyzed by univariate Cox regression analysis, least absolute shrinkage, and selection operator (LASSO) regression analysis, and multivariate Cox regression analysis to identify prognosis-related genes. The developed prognosis model contains ten genes, which are all vital for patient prognosis. The areas under the curve (AUC) values for the developed prognostic model at 1, 3, 5, and 10 years were 0.832, 0.831, 0.880, and 0.857 in the training dataset, respectively. The GSE54467 dataset was used as a validation set to determine the predictive ability of the prognostic signature. Protein–protein interaction (PPI) analysis and weighted gene co-expression network analysis (WGCNA) were used to verify “real” hub genes closely related to the TME. These hub genes were verified for differential expression by immunohistochemistry (IHC) analyses. In conclusion, this study might provide potential diagnostic and prognostic biomarkers for CM.

## Introduction

Cutaneous melanoma (CM), a highly aggressive malignancy, represents approximately 2% of skin cancers and approximately 75% of skin cancer deaths due to rapid progression and metastasis (1). Surgical resection is the optimal treatment option for most early stage melanomas, but limited effective late-stage therapies exist, and only a small proportion of late-stage patients respond to single or combined therapies, limiting patient survival (2). Additional exploration of CM carcinogenesis and treatment remains urgently necessary.

Recently, increasing evidence indicates that the tumor microenvironment (TME) is involved in tumor development. Interactions between cancer cells, stromal cells, and tumor-infiltrating immune cells (TICs) are critical for malignant cancer progression, including the promotion of replicative immortality, invasion, metastasis, and immune surveillance evasion. The TME influences clinical outcomes and contains potential targets for therapeutic modulation (3). Several studies have reported that TICs represent a promising TME index for evaluating therapeutic efficacy (4). TIC components and their activation states are vital parameters that affect patient prognosis and tumor characteristics. Anti-cytotoxic T-lymphocyte antigen 4 (CTLA-4) therapy can activate T cells and induce programmed-death ligand 1(PD-L1) expression in tumor cells and TICs. In many cancers, including CM, CD8^+^ T cell activation can prolong patients’ survival times (5). A study indicated that increased CD8^+^ T cell trafficking contributes to anti-programmed-death 1 (PD-1)/CTLA-4 therapeutic efficacy against melanoma metastasis and may represent an effective immunotherapy strategy (6). Neutrophils also play a context-dependent role in melanoma and can actively switch to an anti-tumor mode (7). These studies suggested that crosstalk between cancer cells and the TME plays an indispensable part in CM development, which has made the accurate delineation of the dynamic regulatory effects of immune and stromal components on the TME challenging.

In the present study, the proportions of immune and stromal components and the TIC ratio were quantified based on the ESTIMATE and CIBERSORT computational methods in skin CM (SKCM) samples obtained from The Cancer Genome Atlas (TCGA) database. Differentially expressed genes (DEGs) were identified in the high-ImmuneScore and high-StromalScore groups compared with the corresponding low-score groups. We utilized patient survival information obtained from TCGA to perform univariate Cox regression, least absolute shrinkage and selection operator (LASSO) regression, and multivariate Cox regression analyses to verify prognosis-related genes. The 79 SKCM samples from the GSE54467 dataset were used as a validation set to verify the predictive ability of the prognostic model. Additionally, we defined a protein–protein interaction (PPI) network based on the identified DEGs to verify hub genes. DEGs in the TCGA database were also examined by weighted gene co-expression network analysis (WGCNA) to identify hub genes related to the ImmuneScore and StromalScore of SKCM. Genes identified in both networks were identified as “real” hub genes critical to the TME. These “real” hub genes were verified by examining differential expression using immunohistochemistry (IHC) analyses. These results provided a better understanding of the underlying biological mechanisms of immune-related genes and may improve SKCM prognosis.

## Materials and Methods

### Data Collection and Data Processing

The RNA sequencing (RNA-seq) data of 471 SKCM samples were downloaded from TCGA (https://portal.gdc.cancer.gov/). Corresponding clinical information was obtained from the UCSC Xena database (http://xena.ucsc.edu/). We used the fragments per kilobase of transcript per million mapped reads (FPKM) method to standardize the data (8). To ensure that significantly expressed genes were evaluated, genes with average expression values <0.1 were excluded from each case. P-values of DEGs were identified using a Wilcox test. Genes with fold change (FC) >1 (high- and low-score groups) and false discovery rate (FDR) <0.05 after log_2_ transformation were regarded as DEGs.

To increase robustness, RNA-seq data and clinical information from an independent cohort of 79 tumor samples were obtained from the GSE54467 dataset (https://www.ncbi.nlm.nih.gov/geo/query/acc.cgi?acc=GSE54467) as a validation set. Processed expression data were log_2_ transformed before further analysis. When multiple probes corresponded to the same gene, the average of all probes was used. Data normalization and background adjustments were conducted using the “limma” R package.

A total of 80 samples were collected from CM patients who underwent surgical resection at the Third Affiliated Hospital of Kunming Medical University from January 2013 to December 2016. CM and adjacent normal tissues were obtained as formalin-fixed paraffin-embedded (FFPE) samples. The study was approved by the Ethics Committee of the Third Affiliated Hospital of Kunming Medical University. The clinical materials and outcome data were reviewed after approval was obtained from the institutional review board.

### Generation of the ImmuneScore, StromalScore, and ESTIMATEScore

The proportions of immune-stromal TME components were quantified for each patient using the ESTIMATE R package. The algorithm includes the ImmuneScore, StromalScore, and ESTIMATEScore, which positively correlate with proportions of immune components, stromal components, and both, respectively, with higher scores indicating increased proportions in the TME (9).

### Functional Enrichment Analysis

The Gene Ontology (GO) analysis consists of biological processes (BPs), cellular components (CCs), and molecular functions (MFs) (10). The Kyoto Encyclopedia of Genes and Genomes (KEGG) database is used for the functional annotation, systematic analysis, and visualization of gene functions (11). GO functional annotations and KEGG enrichment analyses were used to understand the potential biological significance of genes using the clusterProfiler package in R. We listed the top 10 terms in every category, limited to those terms with both p- and q-values <0.05.

### Risk Score System Establishment

Patients’ clinical information was downloaded from the UCSC Xena database. After removing samples without survival data, 454 samples remained for follow-up survival analysis. We randomly divided the samples into training (227 samples) and test (227 samples) groups to ensure the generalizability of the prognostic signature. Univariate Cox proportional hazards regression analysis was performed on the training cohort, with P <0.01 designated as significant, and significant variables were integrated into the LASSO regression analysis (12). To produce the minimum cross-validation error, LASSO regression analysis was used to generate a generalized linear model with 10-fold cross-validation (13). A multivariate Cox proportional hazard regression model, based on the two-step method, was generated to verify key genes involved in the prognostic model. Ten immune-related genes and their corresponding coefficients were used to generate the prognosis model for SKCM. The risk score for each patient was calculated as follows:

$$Riskscore={\text{expr}}_{gene1}*\beta_{gene1}+{\text{expr}}_{gene2}*\beta_{gene2}+{\text{expr}}_{gene3}*\beta_{gene3\ldots \ldots \ldots }{\text{expr}}_{genen}*\beta_{genen}$$

where expr represents the selected gene expression level, and *β* represents the regression coefficients of the multivariate Cox regression model (14). A risk score was calculated for each sample included in this study. Patients were stratified into high- and low-risk groups, according to the median risk value.

### CIBERSORT Estimation

The relative proportions of 22 immune cell types were calculated in each SKCM sample based on the expression file, as assessed by CIBERSORT (15). TIC abundance profiles for all tumor samples were estimated using CIBERSORT. Only the 260 tumor samples with P <0.05 in the CIBERSORT analysis were considered eligible for subsequent analyses.

### PPI Network Construction and Module Analysis

A PPI network can identify hub genes and gene modules according to the level of interaction. The Search Tool for The Retrieval of Interaction Genes (STRING, https://string-db.org/) is an online tool for analyzing consensus genes and constructing PPI networks (16). DEGs were submitted to the STRING database to evaluate PPI information, and nodes with interaction scores >0.95 were selected for PPI network construction. The PPI network was visualized using Cytoscape 3.7.0 software. The top 30 genes, according to the number of nodes, were designated hub genes in the PPI analysis. The biological significance of gene modules was visualized with the plug-in Molecular Complex Detection (MCODE) in Cytoscape to identify the most significant module (17).

### Co-expression Network Construction of DEGs

The WGCNA package in R was used to generate a co-expression network of DEGs. Pearson’s correlation analysis was conducted as a similarity measure for all pair-wise genes. The power function Amn = |Cmn|*β* (Cmn = Pearson’s correlation between gene m and gene n; Amn = adjacency between gene m and gene n) allowed for the construction of a weighted adjacency matrix. The soft threshold power (*β*) of the correlation matrix was used to emphasize strong correlations between genes and penalize weak correlations. A *β* value was selected to construct a co-expression network, and the adjacency was transformed into a topological overlap matrix (TOM) to measure the network connectivity of genes (18). To identify genes with expression profiles similar to the gene modules, we used average linkage hierarchical clustering based on TOM dissimilarity measurements, and the minimum number of genes per module was set to the default of 20 (19). Dissimilarities among the module eigengenes (MEs) in the module dendrogram were calculated, and similar modules were merged.

### Identification of Significant Modules and Functional Annotation

Correlations between clinical information and modules were determined using two methods. MEs were used as the principal component for each gene module. Gene significance (GS) scores were calculated to determine correlations among gene expression in the module, defined as the log_10_ transformation of the P-value (GS = logP) for each gene. Module significance (MS) was defined as the average GS in a specific module, representing the correlation between the module and scores. In general, modules with the largest MS values were considered those associated with the scores. To explore the functions of the modules, GO and KEGG enrichment analyses were used to identify the underlying biological significance of module genes. Only terms with both p- and q-values <0.05 were included.

### Finding “Real” Hub Gene and Verification

In this study, we selected two important modules in the co-expression network, and hub genes were defined as those with high module membership (MM), as measured by Pearson’s correlation analysis (weighted correlation 0.8). The hub genes in the module had the highest correlation with the scores (weighted correlation 0.5). A PPI network with a combined interaction score of >0.95 was constructed. The top 30 genes, ordered by the number of nodes, were selected as hub genes in the PPI analysis. Hub genes identified in both the co-expression and PPI networks were regarded as “real” hub genes for subsequent analysis.

First, the GEPIA database (http://gepia.cancer-pku.cn) and the Human Protein Atlas (HPA; http://www.proteinatlas.org/) were used to validate the expression of “real” hub genes between tumor and normal skin tissues in SKCM (20). Then, the differential expression of “real” hub genes was verified in 80 human CM tissues and adjacent tissues analyzed by IHC. In addition, we investigated four genes reported as key targets for immune checkpoint inhibitors: PD-1 and its ligand PD-L1, indoleamine 2,3-dioxygenase 1 (IDO1), and CTLA-4 in cancer (21–23). To determine the possible roles of our “real” hub genes in immune checkpoint blockade (ICB) treatment, we analyzed the correlation between these immune checkpoint inhibitors and our hub genes. The Tumor Immune Estimation Resource (TIMER) (https://cistrome.shinyapps.io/timer/) algorithm was used to explore the correlation between “real” hub genes and immune cell infiltration in SKCM patients (24). All statistical analyses were performed using R software (version 3.6.3). Differences were considered significant at P <0.05.

### IHC

Paraffin-embedded tumor samples and adjacent samples from CM patients were collected, fixed with 10% formalin buffer, dehydrated, and sectioned. IHC staining was performed using rabbit anti-VAV1 antibody (1:100), rabbit anti-ITGB2 antibody (1:200), and rabbit anti-HLA-DRA antibody (1:200). Semiquantitative expression levels were used to determine the extent and intensity of stained tumor cells. The staining intensity was divided into four levels: blank = 0, yellow = 1, dark yellow = 2, and brown = 3. The frequency of positive cells was divided into five levels: 0–5% = 0, 6–25% = 1, 26–50% = 2, 51–75% = 3, and 76–100% = 4. The immune response score was calculated as the stain intensity score multiplied by the frequency of positive cells. All slides were independently evaluated by two pathologists blinded to the patient’s identity and clinical diagnosis.

## Results

### Scores Correlate With Survival and Are Clinically Relevant in SKCM Patients

**Figure 1:**
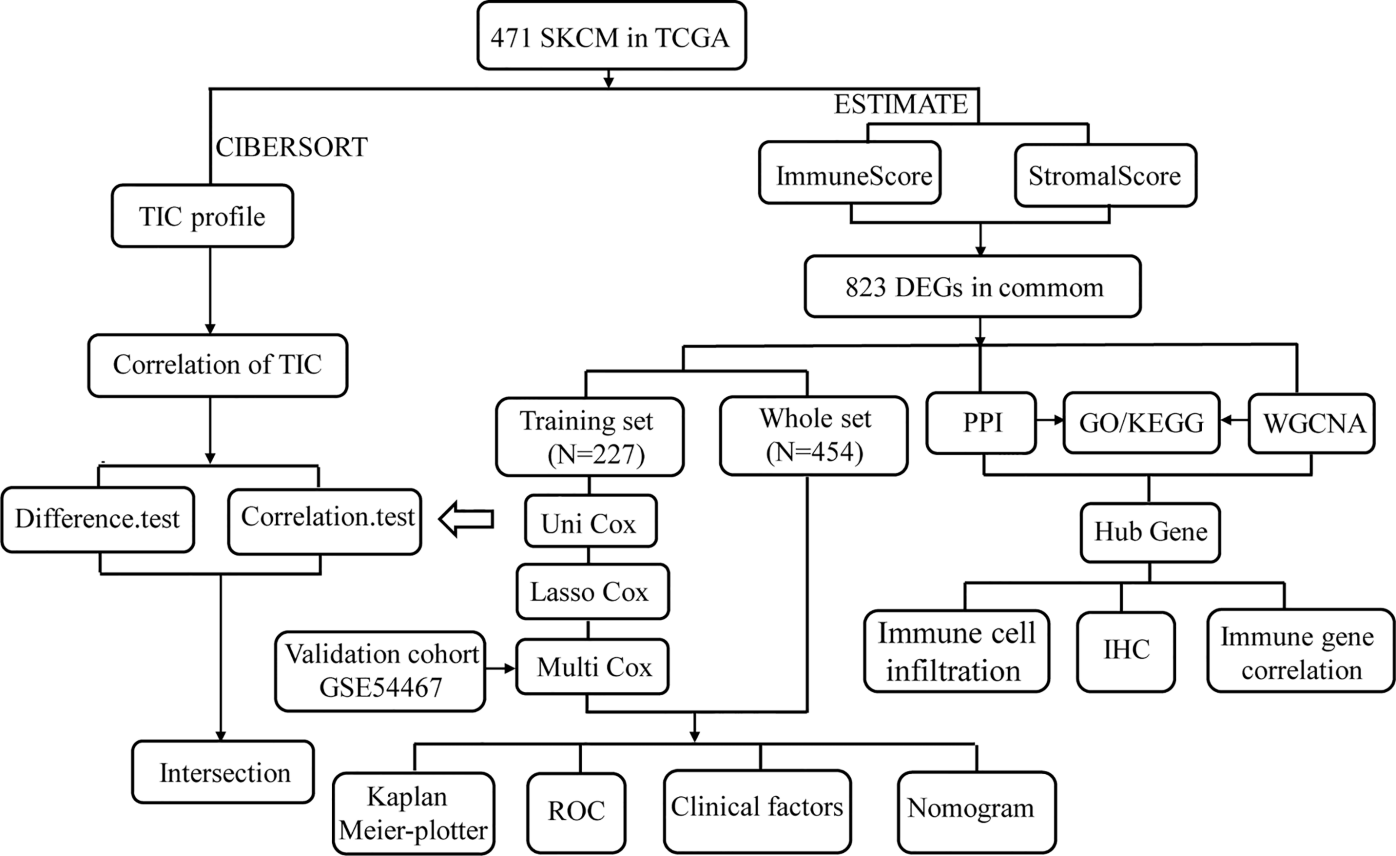
Analysis workflow of this study.

**Figure 2:**
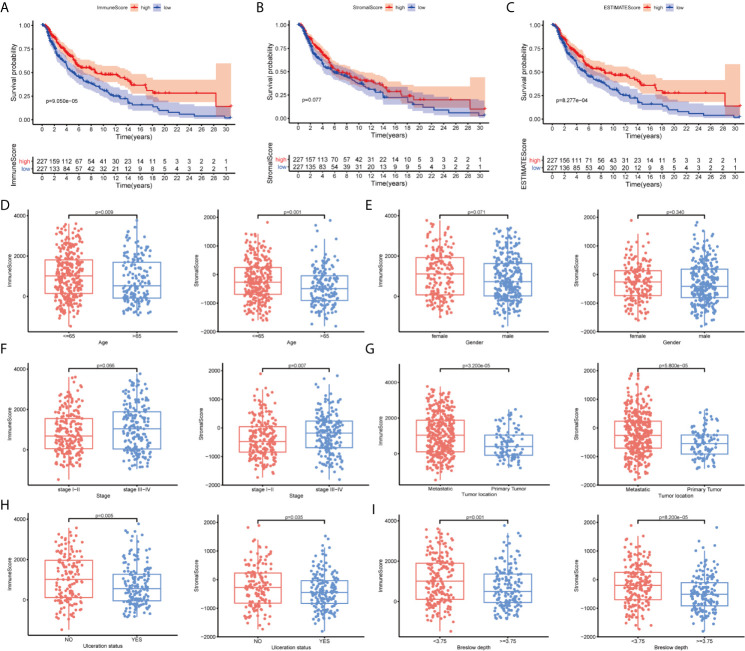
The correlation between scores with clinicopathological features in cutaneous melanoma (CM). **(A–C)** Kaplan–Meier survival analysis identified skin CM (SKCM) patients in the high- or low-ImmuneScore, -StromalScore, and -ESTIMATEScore groups by comparison with the median scores for each analysis. **(D–I)** Correlation between the ImmuneScore and StromalScore and age, sex, tumor node metastasis (TNM) stage, tumor location, ulceration status, and Breslow depth.

In the present study, we systematically analyzed the critical roles and prognostic value of genes related to immune infiltration in SKCM. **Figure 1** shows the overall study design. Correlations between immune and stromal cell proportions and survival rates were determined by grouping melanoma patients into high- and low-score groups, according to the median value of 471 SKCM patients. Kaplan–Meier survival curves were conducted for the ImmuneScore, StromalScore, and ESTIMATEScore. As shown in **Figure 2A**, high-ImmuneScore patients had better survival than low-ImmuneScore patients. Although no significant correlation was found between the StromalScore and overall survival (OS) (**Figure 2B**), the OS was significantly higher among high-ESTIMATEScore patients than low-ESTIMATEScore patients (**Figure 2C**). These results indicated that the proportions of immune components were significant prognosis indicators for SKCM patients.

To clarify the correlation between scores and clinical features, we analyzed the clinical features of SKCM patients from the UCSC Xena database. Older patients had significantly lower scores than younger patients (**Figure 2D**; P = 0.009 and P = 0.001, respectively). Women had higher ImmuneScores and StromalScores than men although not significantly different (**Figure 2E**; P = 0.071 and P = 0.340, respectively). Advanced-stage cases generally had higher scores than early stage cases (**Figure 2F**; P = 0.066 and P = 0.007, respectively), and metastatic tumors had higher scores than primary tumors (**Figure 2G**; P < 0.001). Patients without ulcerations or with lower Breslow depths had higher immune and stromal scores (**Figures 2H, I** **)**. These results indicated that the TME, especially TICs, may play indispensable roles in SKCM progression, although further exploration remains necessary.

### Identification of DEGs Shared by the ImmuneScore and StromalScore

**Figure 3:**
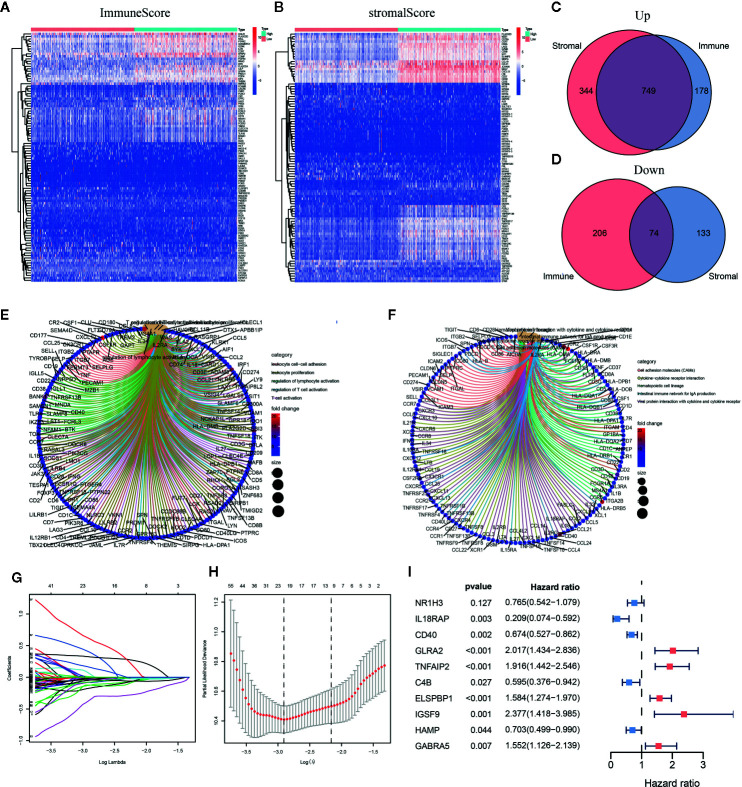
Heatmaps, Venn diagrams, and enrichment analysis of differentially expressed genes (DEGs) and the construction of the prognostic classifier. **(A, B)** Heatmap for DEGs generated by comparing the high-score group *vs.* the low-score group for the ImmuneScore and StromalScore. **(C, D)** Venn diagrams showing common upregulated and downregulated DEGs shared by the ImmuneScore and StromalScore analyses. **(E, F)** Gene Ontology (GO) and Kyoto Encyclopedia of Genes and Genomes (KEGG) enrichment analyses for 823 DEGs. **(G, H)** The number of included factors was determined by LASSO analysis. **(I)** A forest plot showing the hazard ratios (HRs) and P-values from the multivariate Cox regression.

To clarify changes in gene expression levels among immune and stromal components in the TME, we compared high- and low-score samples to identify DEGs. The results indicated that 927 genes were upregulated, and 280 genes were downregulated by comparing the high-score group *vs.* the low-score group for the ImmuneScore. Similarly, 1,093 genes were upregulated, and 207 genes were downregulated by comparing the high-score group *vs.* the low-score group for the StromalScore (**Figures 3A, B** **)**. The identified intersection genes included 749 upregulated and 74 downregulated genes in both the high-ImmuneScore and high-StromalScore groups compared with the low-score groups, as displayed in the Venn diagram (**Figures 3C, D** **)**. The functions of these 823 genes were predicted, and GO analysis was performed, which showed that these genes were primarily associated with immune-related GO terms, such as leukocyte cell–cell adhesion and leukocyte proliferation (**Figure 3E**). The genes were highly enriched in cell adhesion molecules, cytokine–cytokine receptor interactions, and hematopoietic cell lineages, according to KEGG analysis (**Figure 3F**). The gene enrichment analysis indicated that these genes were primarily associated with immune-related pathway activation, suggesting that immune factors play an indispensable role in the TME of SKCM patients.

### Prognosis-Related Model Construction and Analysis

Among the intersecting DEGs, 436 genes were significantly correlated with prognosis by univariate Cox regression analysis (**Supplementary Table 1**). The genes with the highest potential prognostic significance were identified by LASSO regression analysis. Following 10-fold cross-validation, 20 genes remained (**Figures 3G, H** **)**. A multivariate Cox proportional hazards model was generated to build an immune-related prognostic signature based on the LASSO regression analysis (**Figure 3I**). Ten genes (nuclear receptor subfamily 1 group H member 3, *NR1H3*; interleukin 18 receptor accessory protein, *IL18RAP*; *CD40*; glycine receptor alpha 2, *GLRA2*; tumor necrosis factor (TNF) alpha-induced protein 2, *TNFAIP2*; *C4B*; epididymal sperm binding protein 1, *ELSPBP1*; immunoglobulin superfamily member 9, *IGSF9*; hepcidin antimicrobial peptide, *HAMP*; and gamma-aminobutyric acid type A receptor subunit alpha5, *GABRA5*) were identified by the multivariate Cox proportional hazards regression analysis and were used to generate a prognostic signature by calculating a risk score, as follows:

$$\begin{array}{l} Riskscore=(-0.2685\times NR1H3)+(-1.5631\times IL18RAP)\\ \;+(-0.3943\times CD40)+(0.7016\times GLRA2)\\ \;+(0.6504\times TNFAIP2)+(-0.5186\times C4B)\\ \;+(0.4602\times ELSPBP1)+(0.8657\times IGSF9)\\ \;+(-0.3526\times HAMP)+(0.4398\times GABRA5) \end{array}$$

**Figure 4:**
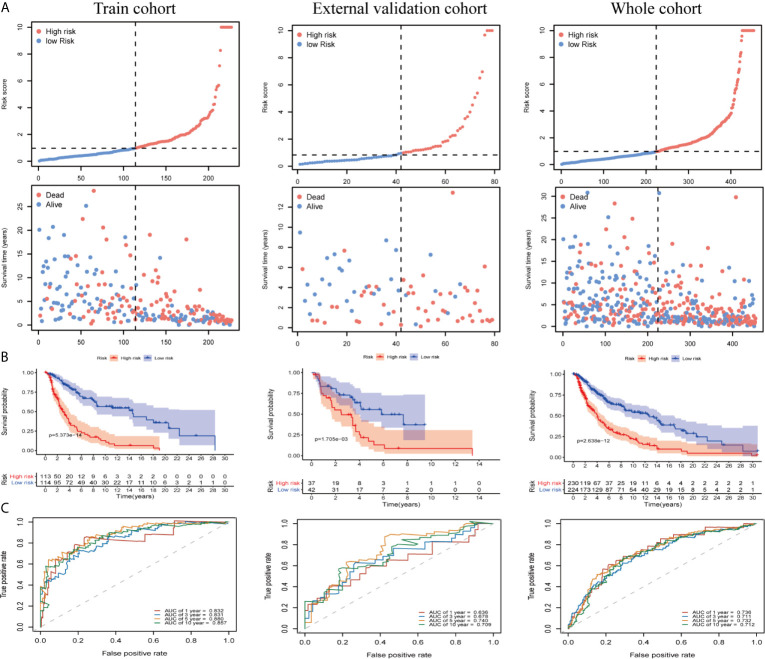
Risk score analyses for the developed immune-related prognostic signature in the training cohort, the external validation cohort, and the whole cohort. **(A)** Patients were ranked by risk score values and the corresponding survival status. **(B)** Kaplan–Meier curves for the immune-related signature. **(C)** Receiver operator characteristic (ROC) curves for survival as predicted by the risk score.

We divided patients into low- and high-risk groups based on the median risk score of the training group. The risk score distribution was ranked according to the risk score values shown in the training cohort, the external validation cohort, and the whole cohort. Patients with a high-risk score had higher mortality than patients with a low-risk score (**Figure 4A**). Consistent with these results, the Kaplan–Meier curves suggested that patients in the low-risk group had higher survival than those in the high-risk group (**Figure 4B**; all P < 0.01). The results showed that the risk scores obtained using the ten-gene prognostic signature predicted survival at 1, 3, 5, and 10 years, with respective AUC values of 0.832, 0.831, 0.880, and 0.857 in the training cohort, 0.636, 0.678, 0.740, and 0.709 in the GSE54467 validation cohort, and 0.736, 0.711, 0.732, and 0.712 in the whole cohort, respectively (**Figure 4C**). These results indicated the high sensitivity and accuracy of the ten-gene prognostic signature in CM.

**Figure 5:**
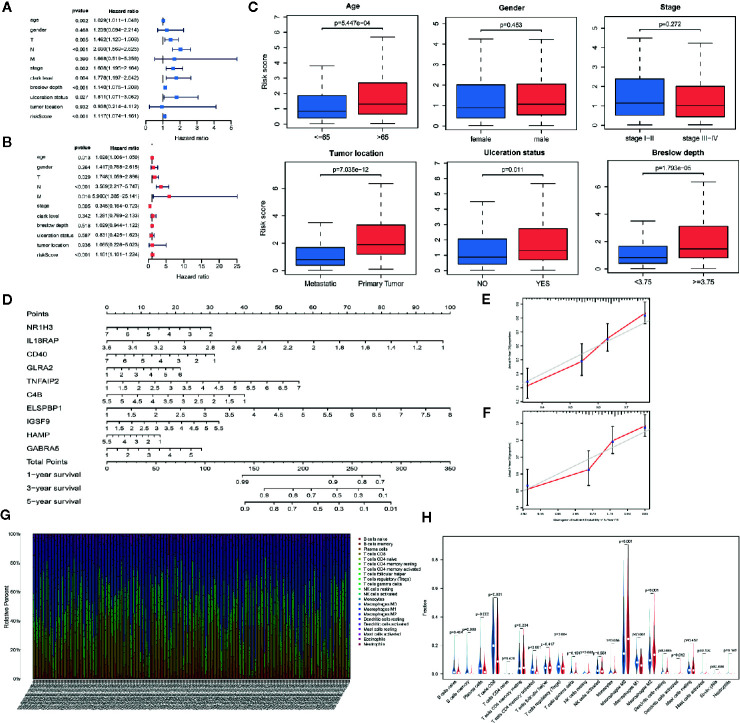
The analysis of the prognosis-related model. **(A, B)** Univariable and multivariable analyses based on the risk group and other clinical features in the TCGA cohort. **(C)** Box plots displaying the correlation between different clinical features and the risk score. **(D)** Nomogram showing the overall survival (OS) for 1, 3, and 5 years in the TCGA cohort. **(E, F)** Calibration plots of the nomogram for predicting OS at 3 and 5 years. **(G)** Bar plot showing the ratios of 22 tumor-infiltrating immune cells (TIC) types in skin cutaneous melanoma (SKCM) patients. **(H)** Violin plot showing the proportions of 22 types of TICs in SKCM patients with low- or high- risk scores relative to the median risk score.

We evaluated the independent prognostic value of our ten-gene model (**Figure 5A**). The risk score was analyzed in combination with age, sex, tumor node metastasis (TNM) stage, tumor location, ulceration status, and Breslow depth, which are closely related to patient survival. Multivariate Cox regression analysis indicated that the ten-gene model is a robust and independent prognostic factor in the whole cohort (P < 0.001, **Figure 5B**). Although only age, sex, and TNM stage data were available for GSE54467, we also tested the validation dataset, which demonstrated consistent results (**Supplementary Figures 1A, B** **)**. The correlation between our prognostic signature and clinical SKCM characteristics was evaluated for the whole cohort (**Figure 5C**). The results indicated that our prognostic model was not associated with sex or TNM stage but was significantly correlated with age (P < 0.001), tumor location (P < 0.001), ulceration status (P = 0.011), and Breslow depth (P < 0.001) in CM, suggesting that the genes in our prognostic model may play essential roles in CM progression.

To build a quantitative model for survival probability prediction in SKCM, we used the ten-gene marker to develop a nomogram plot for estimating the survival probability after 1, 3, and 5 years in the TCGA cohort (**Figure 5D**). The nomogram performance was visualized intuitively by drawing calibration plots, which indicated that the prediction results were consistent with the observed results (**Figures 5E, F** **)**.

### Correlation Between Risk Score and TIC Proportions

The TIC subsets in the TME were quantified according to the CIBERSORT algorithm to determine correlations with the risk score. The abundances of 22 immune cell types in SKCM patients were obtained (**Figure 5G**). Nine TIC types were associated with low- and high-risk groups (**Figure 5H**, **Supplementary Figures 1C, D** **)**, including three TIC types positively correlated with the risk score: M0 macrophages, M2 macrophages, and activated dendritic cells. Memory B cells, plasma cells, CD8^+^ T cells, CD4-activated memory T cells, regulatory T cells (Tregs), and M1 macrophages were negatively correlated with the risk score. These results demonstrated that the risk score might serve as an immune activity indicator.

### PPI Network Analysis of DEGs

**Figure 6:**
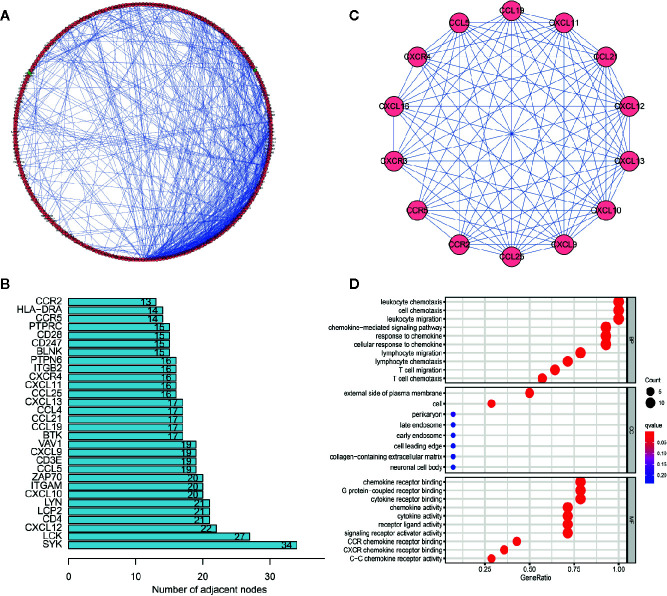
Protein–protein interaction (PPI) network and module analysis. **(A)** PPI network of differentially expressed genes (DEGs). **(B)** The top 30 genes were ranked by the number of nodes. **(C)** PPI network of the top significant module. **(D)** Gene ontology (GO) analysis of the top significant module in the PPI analysis.

To determine the hub genes and relevant gene modules involved in SKCM, we built a PPI network for the DEGs using Cytoscape software based on data obtained from the STRING database. The network consisted of 282 nodes and 746 edges (**Figure 6A**). The top 30 genes according to the number of nodes were displayed in a bar plot (**Figure 6B**). The top significant module was identified by the plug-in MCODE in Cytoscape (**Figure 6C** **)**. Functional and pathway enrichment analyses of the DEGs in the top module were performed. The GO analysis showed that DEGs in the top module were involved in the leukocyte chemotaxis and cell chemotaxis in BPs. The CC analysis indicated genes enriched on the external side of the plasma membrane. The MF analysis showed genes enriched in G protein-coupled receptor binding and chemokine receptor binding (**Figure 6D**). KEGG analysis revealed DEGs principally involved in the chemokine signaling pathway (**Supplementary Figure 2A**).

### WGCNA of DEGs

**Figure 7:**
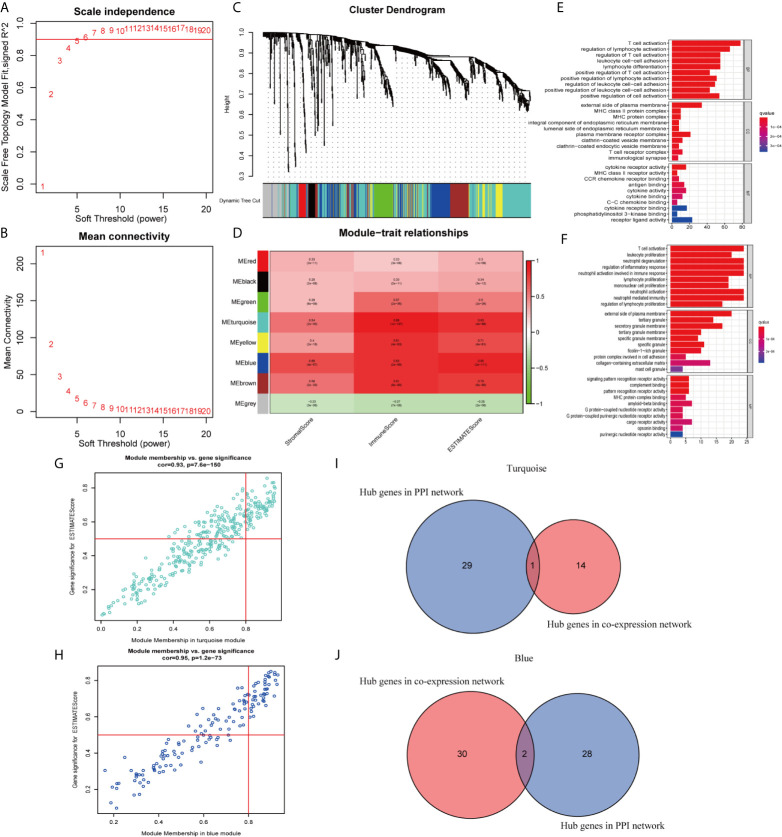
Modules related to skin cutaneous melanoma (SKCM) scores and hub gene detection. **(A, B)** Analysis of the scale-free fit index and the mean connectivity for various soft-thresholding powers. **(C)** Dendrogram of all differentially expressed genes (DEGs) clustered based on a dissimilarity measure. **(D)** A heatmap showing the correlation between the gene modules and scores. **(E, F)** Gene ontology (GO) analyses of the turquoise and blue modules. **(G, H)** Scatter plot of the module eigengenes (MEs) in the turquoise and blue modules. **(I, J)** Identification of “real” hub genes in the protein–protein interaction (PPI) network and the co-expression network in the turquoise and blue modules.

In this study, 471 scored SKCM samples were used for co-expression analysis. The soft-thresholding power was set to 5 to generate a scale-free network (**Figures 7A, B** **)**. A total of eight modules were verified based on the SKCM scores (**Figure 7C**). Module-trait correlation analyses showed the turquoise and blue modules with the highest score associations (**Figure 7D**). GO (**Figures 7E, F** **)** and KEGG (**Supplementary Figures 2B, C**) enrichment analyses indicated that the turquoise module was principally concentrated in T cell activation, regulation of lymphocyte activation, and regulation of T cell activation, whereas the blue module was associated with T cell activation, leukocyte proliferation, and neutrophil degranulation. The genes in the blue and turquoise modules were pivotal for immune cell infiltration.

### Hub Genes Related to TICs in SKCM

We identified 15 genes closely related to immune function in the turquoise module and 32 genes in the blue module as candidate hub genes (**Figures 7G, H** **)**. The shared genes between the top 30 PPI nodes and the turquoise and blue modules were identified. Vav guanine nucleotide exchange factor 1 (*VAV1*) in turquoise, integrin subunit beta 2 (*ITGB2*) and major histocompatibility complex, class II, DR alpha (*HLA-DRA*) in blue were identified as candidates for further analysis and validation (**Figures 7I, J** **)**. These genes were defined as “real” hub genes associated with TICs in SKCM.

**Figure 8:**
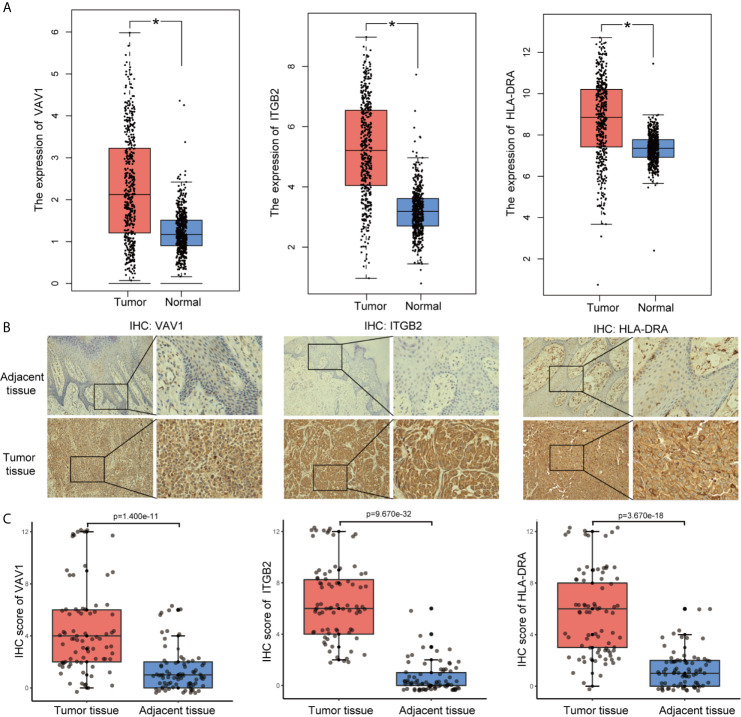
Validation of “real” hub genes. **(A)** Hub gene expression in CM and normal skin tissues, based on the GEPIA database. **(B)** Examples of immunohistochemistry (IHC) staining for hub genes in CM tissues and corresponding adjacent tissues. **(C)** The expression of hub genes in 80 human CM tissues and adjacent tissues analyzed by IHC.

**Figure 9:**
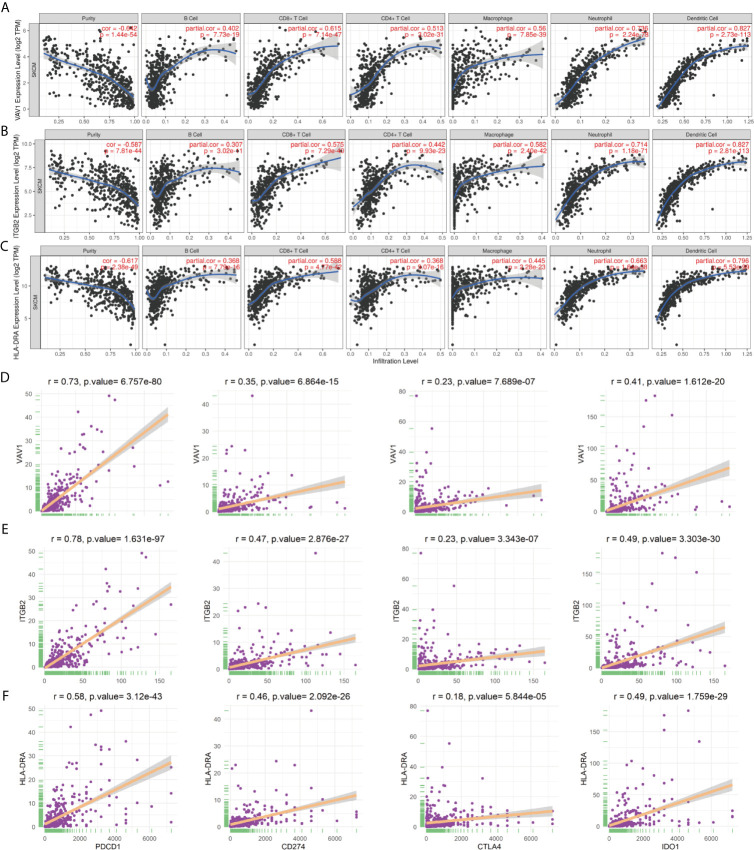
“Real” hub genes were related to immune infiltration processes. **(A–C)** Correlation between immune cell abundance and hub gene expression. “Purity” represents the purity of the tumor cells in the sample. **(D–F)** The correlation between hub genes and the immune checkpoint inhibitor targets PD-1, PD-L1, CTLA-4, and IDO1.

To identify the roles played by “real” hub genes in SKCM, we first used the GEPIA and the HPA database to compare “real” hub gene expression between CM and normal skin tissues. The results showed that the three hub genes were significantly upregulated in tumor tissues compared with normal skin (**Figure 8A**, **Supplementary Figures 3A–C**). Then, we performed IHC analyses to determine and compare the expression levels of hub genes in 80 human CM and adjacent tissues. Representative images of IHC staining for the hub genes are shown in **Figure 8B**. According to IHC staining results, we measured the expression of hub genes in 80 CM and adjacent tissues. The results indicated that the expression of the three hub genes significantly higher in tumor tissues than in adjacent tissues (**Figure 8C**). To understand the functions of “real” hub genes, we investigated the correlations between hub genes and immune infiltration using the TIMER database. There was a positive correlation between hub gene expression and the immune cell infiltration in SKCM (**Figures 9A–C**). The expression of immune checkpoint genes may be associated with the therapeutic efficacy of immune checkpoint inhibitors (25). ICB tumor immunotherapy has advanced in recent years, including for CM (22, 26). We evaluated the association between four key ICB targets and the “real” hub genes: PD-1, PD-L1, CTLA-4, and IDO1 (21–23). We found that *VAV1* was positively related to PD-1 (r = 0.73; P < 0.001), PD-L1 (r = 0.35; P < 0.001), CTLA4 (r = 0.23; P < 0.001), and IDO1 (r = 0.41; P < 0.001) (**Figure 9D**). Similar results were obtained for *ITGB2* and *HLA-DRA* (**Figures 9E, F** **)**, suggesting that these hub genes may play significant roles in the responses to ICB immunotherapy in SKCM. These results indicated that these three genes play significant roles in immune infiltration processes in SKCM patients and may represent potential therapeutic targets.

## Discussion

The CM incidence and mortality have increased recently, which is a public issue that attracts worldwide attention. Despite many studies on SKCM, early diagnosis, treatment, and prognosis remain poor. Investigating the potential molecular biological mechanisms underlying SKCM progression and development is important. Recently, many advanced therapeutic options have been developed for melanoma patients, improving disease-free rates and OS. However, limitations persist, including low sustained response rates, drug toxicity, low tolerance, high cost, and patient responses are heterogeneous (27). The rapid development of high-throughput sequencing technology facilitates the detection of abnormal gene expression during tumor progression, providing effective targets for diagnosis and treatment. A lack of reliable biomarkers exists to monitor therapeutic efficacy. Therefore, we attempted to identify genes that affect patient prognosis by investigating the TME.

Increasing evidence suggests the TME is a vital modulator of tumor progression, and the identification of potential therapeutic targets associated with TME remodeling can promote the TME transformation from tumor-supportive to tumor-suppressive. Transcriptome analysis of SKCM data from the TCGA and Gene Expression Omnibus (GEO) databases demonstrated that the proportions of immune and stromal components in the TME had important influences on SKCM progression. Our results emphasized the importance of interactions between tumor and immune cells, providing new insights into SKCM immunotherapy. Despite recent achievements in ICB-based tumor immunotherapy for advanced SKCM patients (28, 29), fewer than one-third of patients treated with ICB achieve good therapeutic effects. Immune checkpoint gene expression cannot accurately predict ICB treatment efficacy. Therefore, biomarkers capable of predicting the ICB immunotherapy response are essential (30).

In this study, we generated an immune-related prognostic model to predict the patient survival rate, which consisted of ten genes: *NR1H3*, *IL18RAP*, *CD40*, *GLRA2*, *TNFAIP2*, *C4B*, *ELSPBP1*, *IGSF9*, *HAMP*, and *GABRA5*. Some genes in the model have previously been associated with the formation and regulation of the TME. For example, *NR1H3* belongs to the NR1 subfamily of nuclear receptors, which are vital regulators of macrophage function and transcription processes during inflammation (31). Related studies have demonstrated that *NR1H3* can impair the anti-tumor response by inhibiting the CCR7 expression on dendritic cells, suggesting a novel mechanism for immune escape (32). In addition, the *NR1H3-*mediated promotion of the epithelial-mesenchymal transition (EMT) and migration of tumor cells has been reported in several cancers (33, 34). *IL18RAP* also modulates the TME and impacts cancer progression through proinflammatory functions. *ILI8RAP* is an accessory subunit of the heterodimeric interleukin 18 (IL18) receptor (35). CD40 is a member of the TNF receptor superfamily (36). In mouse melanoma, tumor endothelial cells upregulate *IDO1* in response to the increased secretion of interferon (IFN)*γ* by CD40-stimulated immunotherapy, revealing a new immunosuppressive feedback mechanism (37). Immunotherapy success in CM depends on the activation of functional T cells in the tumor. Singh et al. showed that locally focused ultrasound (FUS) heating combined with *in situ* anti-CD40 agonist antibody improved T cells and macrophage function, promoting effective melanoma immunotherapy (38). *TNFAIP2* expression can be induced by TNFα. The abnormal expression of *TNFAIP2* has been identified in various malignant tumors, involved in unlimited proliferation, angiogenesis, and migration, including urothelial cancer, esophageal squamous cell carcinoma, and nasopharyngeal carcinoma (39–42). Although some biological functions of these ten genes have not previously been reported in SKCM, their roles in progression and tumor immunity require further study. We indicate that the ten-gene prognosis model can be used as an indicator of the SKCM immunotherapy response.

By combining the PPI and WGCNA, three genes were verified as “real” hub genes associated with the TME in SKCM. Based on correlations between genes, we construct a WGCNA network, and a PPI network was generated based on available literature. The combination of the WGCNA and PPI methods appears to be suitable for hub gene identification. Several studies have indicated the abnormal expression of hub genes in various malignant tumors, which may represent important prognostic biomarkers. *VAV1* is a member of the *VAV* gene family and is vital for hematopoiesis, which plays an indispensable role in T cell and B cell activation (43). Related studies have indicated that IDO can inhibit the T cell response and promote immune tolerance by downregulating *VAV1* expression and inhibiting the *VAV1*/Rac cascade reaction (44). *ITGB2* encodes integrin beta chain, a cell surface protein involved in cell adhesion and cell surface-mediated signal transduction. *ITGB2* plays a significant role in the immune response, and *ITGB2* deficiency causes leukocyte adhesion defects (45, 46). A prospective study revealed that high *ITGB2* expression in cancer-associated fibroblasts promoted tumor proliferation in oral squamous cell carcinoma through NADH oxidation in the mitochondrial oxidative phosphorylation system (47). Another study showed that *ITGB2* downregulated Treg cells levels and inhibited renal carcinoma development (48). *HLA-DRA* is an HLA class II alpha chain paralog that plays a vital role in the immune system and responses by presenting peptides. *HLA-DRA* is highly expressed in bladder cancer tissues than corresponding adjacent tissues and indicates poor progression-free survival (49). In kidney renal clear cell carcinoma, *HLA-DRA* serves as a reliable biomarker and may play a vital role in cancer immunotherapy (50). A recent clinical trial showed that *HLA-DRA* predicted the advanced melanoma immune response to tremelimumab, which blocks CTLA-4 (51).

However, this study has some limitations. First, there is no detailed clinical data on the treatment of patients in TCGA and GEO databases, although other clinical factors available from the databases have been included. Second, analysis based on transcriptomics can represent only certain aspects of the immune microenvironment but not the overall process of change. In addition, further experimental studies are needed to elucidate the potential mechanism of the prognostic model and hub genes in the occurrence and development of CM.

## Conclusion

We successfully constructed a prediction model with good accuracy. Differences in OS between high- and low-risk groups were associated with immune cell infiltration and the complex regulation of multiple signaling pathways. By combining the PPI and WGCNA network analyses, “real” hub genes closely related to the TME were identified. Our study provides additional supplementary insights for analyzing the pathogenesis and response to immunotherapy of CM.

## Data Availability Statement

Publicly available datasets were analyzed in this study. The datasets generated for this study can be found here: The Cancer Genome Atlas (TCGA) (https://portal.gdc.cancer.gov/) and Gene Expression Omnibus (GEO) datasets (https://www.ncbi.nlm.nih.gov/geo/query/acc.cgi?acc=GSE54467).

## Ethics Statement

The study involving human participants was reviewed and approved by the Third Affiliated Hospital of Kunming Medical University. The patients/participants provided their written informed consent to participate in this study.

## Author Contributions

RQ and WP were responsible for the study design, data acquisition, and analysis and were major contributors to writing the manuscript. XW, CL, and YX helped to perform the data analysis. CS and ZZ were responsible for the integrity of the entire study and manuscript review. All authors contributed to the article and approved the submitted version.

## Funding

This work was supported by grants from the National Natural Science Foundation of China (81560470, 81773127 and 81960543), Special Funds for Innovation Team of Basic and Clinical Research of Head and neck Tumor in Yunnan Province; Special Funds for High-Level Medical Leaders in Yunnan Province (L−2017025), and Yunnan Province Basic Research Program (2018FE001‑058/246, 2019FE001-075 and 202001AY070001-024).

## Conflict of Interest

The authors declare that the research was conducted in the absence of any commercial or financial relationships that could be construed as a potential conflict of interest.
