# Supplementary material for: Identification of Genes Related to Immune Infiltration in the Tumor Microenvironment of Cutaneous Melanoma
Source: Front Oncol. 2021 May 28;11:615963. doi: 10.3389/fonc.2021.615963 (PMC8202075; doi:10.3389/fonc.2021.615963)
Supplement: Supplementary file 1 [file DataSheet_1.docx]

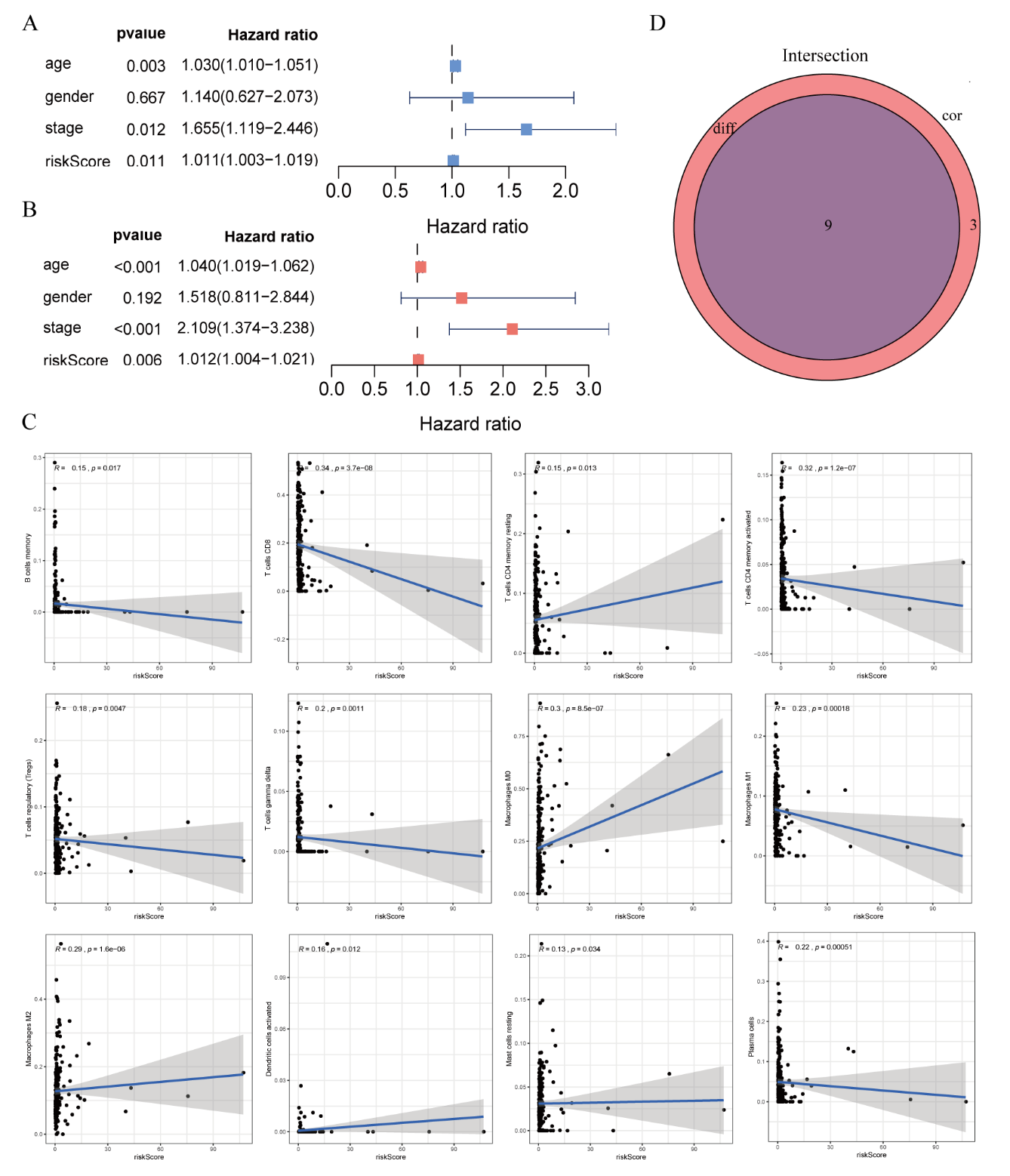


**Supplementary Figure 1**. Analysis of the prognosis-related model. (A, B) Univariable and multivariable analyses according to the risk group and other clinical features in the GSE54467 dataset. (C) Scatter plot showing the correlation between the proportions of 12 tumor-infiltrating immune cell (TIC) types and risk scores (P < 0.05). (D) Venn diagram showing the risk score associated with the nine TIC types determined by difference and correlation tests, which are displayed as violin and scatter plots, respectively.
