# Supplementary material for: Identification of Genes Related to Immune Infiltration in the Tumor Microenvironment of Cutaneous Melanoma
Source: Front Oncol. 2021 May 28;11:615963. doi: 10.3389/fonc.2021.615963 (PMC8202075; doi:10.3389/fonc.2021.615963)
Supplement: Supplementary file 2 [file DataSheet_2.docx]

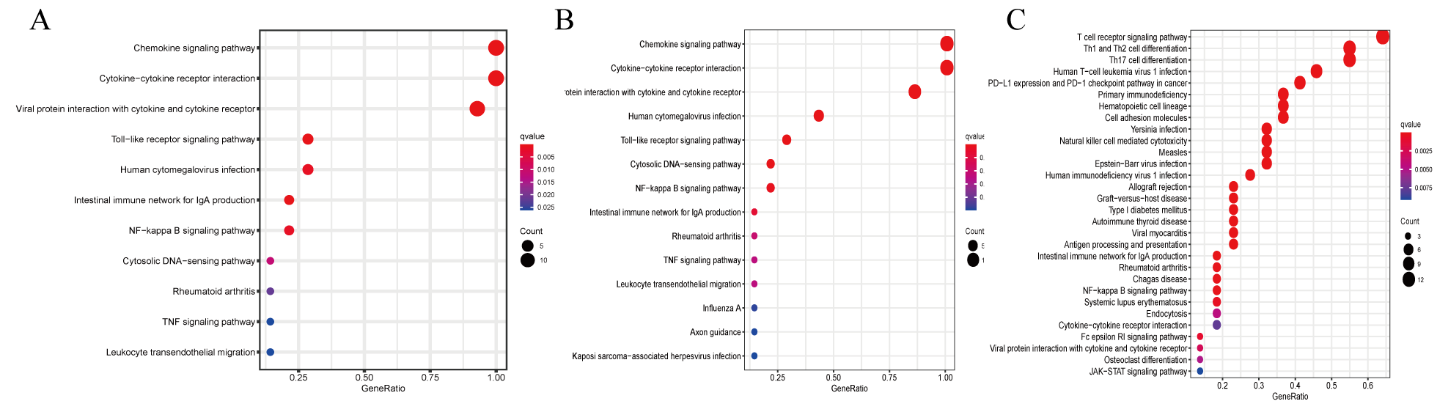


**Supplementary Figure 2.** (A) Kyoto Encyclopedia of Genes and Genomes (KEGG) analysis of the top module in the PPI analysis. (B, C) KEGG analysis in the turquoise and blue modules.
