# Supplementary material for: Identification of Genes Related to Immune Infiltration in the Tumor Microenvironment of Cutaneous Melanoma
Source: Front Oncol. 2021 May 28;11:615963. doi: 10.3389/fonc.2021.615963 (PMC8202075; doi:10.3389/fonc.2021.615963)
Supplement: Supplementary file 3 [file DataSheet_3.docx]

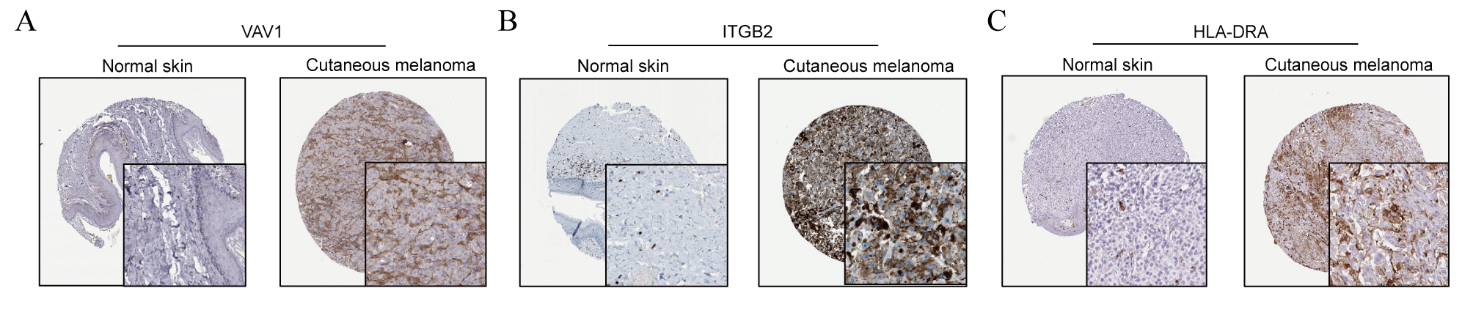


**Supplementary Figure 3**. (A–C) The identification of hub gene expression at the translational level, according to the HPA database.
